# Dynamic balancing responses in unilateral transtibial amputees following outward-directed perturbations during slow treadmill walking differ considerably for amputated and non-amputated side

**DOI:** 10.1186/s12984-021-00914-3

**Published:** 2021-07-31

**Authors:** Andrej Olenšek, Matjaž Zadravec, Helena Burger, Zlatko Matjačić

**Affiliations:** University Rehabilitation Institute, Linhartova 51, 1000 Ljubljana, Slovenia

**Keywords:** Unilateral transtibial amputation, Dynamic balancing response, Perturbed walking, In-stance strategy, Stepping strategy, Center of mass, Center of pressure, Ground reaction force

## Abstract

**Background:**

Due to disrupted motor and proprioceptive function, lower limb amputation imposes considerable challenges associated with balance and greatly increases risk of falling in presence of perturbations during walking. The aim of this study was to investigate dynamic balancing responses in unilateral transtibial amputees when they were subjected to perturbing pushes to the pelvis in outward direction at the time of foot strike on their non-amputated and amputated side during slow walking.

**Methods:**

Fourteen subjects with unilateral transtibial amputation and nine control subjects participated in the study. They were subjected to perturbations that were delivered to the pelvis at the time of foot strike of either the left or right leg. We recorded trajectories of center of pressure and center of mass, durations of in-stance and stepping periods as well as ground reaction forces. Statistical analysis was performed to determine significant differences in dynamic balancing responses between control subjects and subjects with amputation when subjected to outward-directed perturbation upon entering stance phases on their non-amputated or amputated sides.

**Results:**

When outward-directed perturbations were delivered at the time of foot strike of the non-amputated leg, subjects with amputation were able to modulate center of pressure and ground reaction force similarly as control subjects which indicates application of in-stance balancing strategies. On the other hand, there was a complete lack of in-stance response when perturbations were delivered when the amputated leg entered the stance phase. Subjects with amputations instead used the stepping strategy and adjusted placement of the non-amputated leg in the ensuing stance phase to make a cross-step. Such response resulted in significantly larger displacement of center of mass.

**Conclusions:**

Results of this study suggest that due to the absence of the COP modulation mechanism, which is normally supplied by ankle motor function, people with unilateral transtibial amputation are compelled to choose the stepping strategy over in-stance strategy when they are subjected to outward-directed perturbation on the amputated side. However, the stepping response is less efficient than in-stance response.

## Background

Dynamic stability during walking is sustained and falls averted if proper regulation of center-of-mass (COM) and whole-body angular momentum are continuously maintained, particularly in the frontal plane where individuals with lower limb amputation are most unstable [14, 29, 36]. Maintaining dynamic stability during walking is a well-documented challenge in persons with Unilateral Transtibial Amputation (UTA) and is associated with the loss of ankle efferent input and sensory information. It is estimated that approximately half of community-living persons with lower limb amputation fall each year due to impaired balance [21, 30]. To strengthen dynamic stability during walking they prolong support on their non-amputated side, their steps are wider and faster and walk slower than persons without amputation [2, 13, 23, 34, 37] and they tend to increase the input from hip muscles to compensate for the lack of ankle muscle power [35, 39]. Such gait is not effective as it increases metabolic costs [5, 8] and leads to other unwanted physical conditions such as back pain [10, 41].

If walking conditions are challenging additional proactive or reactive gait adaptations are needed. When UTA subjects walk on destabilizing walking surface they display proactive adaptation that manifest in decreased step lengths, increased cadence and even wider steps [2, 7, 11, 13]. Similar proactive adaptations in gait parameters were observed also when subjects without amputations walked on destabilizing walking surface [2, 7, 13]. Unlike proactive gait adaptations reactive responses of UTA subjects have been addressed in the literature less extensively. Experiments where perturbing pushes were delivered to the pelvis in the frontal plane [23, 24] showed that UTA subjects use proactive adaptations only if the timing of perturbation is predictable and when perturbation is delivered on their amputated side [23]. They also showed that if perturbation timing is known peak COM displacement is smaller [24]. However if the timing of perturbation is not known in advance no proactive adaptations took place and unexpected perturbations had to be negotiated purely through reactive feedback mechanisms [23, 24]. Similar studies where UTA subjects were subjected to unexpected medio-lateral foot placement perturbation showed an important role of hip strategies in recovering balance [29].

These studies explored dynamic balancing responses of UTA subjects at relatively high walking speed: 0.8 m/s [23, 24] and 1.2 m/s [2, 13, 29]. To the best of our knowledge we are not aware of any study that has investigated dynamic balance of UTA subjects at slow walking, e.g. 0.5 m/s, which is common speed of walking of UTA subjects after discharge [1]. Therefore it is not known to what extent the results of these studies apply also to dynamic balancing responses of UTA subjects if they walk slowly in challenging conditions. So far the effect of low walking speed on dynamic balancing responses has been explored only in subjects without amputations [25, 27, 42]. These studies have shown that when subjects without amputations are subjected to unexpected inward perturbations to the pelvis reactive responses are fairly uniform across walking speeds and perturbation parameters [27] and consist primarily of adequate placement of ensuing step(s), which has been termed as the “stepping” strategy [4, 27]. Similar strategy is applied after unexpected outward-directed perturbation but only when the walking speed is relatively high (above 0.8 m/s). However if walking speed is low (0.4 to 0.6 m/s) at the time of unexpected outward-directed perturbation “in-stance” strategy plays the dominant role [27]. “In-stance” strategy is composed of: “medio-lateral ankle strategy”, characterized with lateral displacement of COP in the direction of perturbation; “braking strategy”, characterized with anterior displacement of COP resulting in braking of COM in the plane of progression; and “inertial strategy”, characterized with impulse-like increase of lateral component of ground-reaction-force (GRF) that opposes the action of perturbation [28]. We have shown in our recent study in subjects without disabilities that a delicate interplay of all three strategies is required to adequately control whole-body angular momenta in the sagittal and frontal planes [28]. Control of COP is predominantly under the control of ankle muscles, which are resources missing in the UTA population and the modulation of lateral GRF is predominantly under the control of hip muscles, which are still at disposal after below-knee amputation. Due to lack of studies it is currently not known what kinematic or kinetic changes UTA subjects resort to if unexpected outward-directed perturbation is applied to their pelvis during slow walking.

The aim of this study was to investigate the kinematics and kinetics of dynamic balancing responses that UTA subjects use after unexpected outward-directed perturbing pushes to the pelvis while walking slowly on a treadmill. Our hypothesis was that the absence of ankle efferent and afferent function in UTA subjects, limiting their abilities to properly modulate COP and GRF under the prosthesis when perturbation commences while the amputated side is in-stance, would make use of “in-stance” balancing strategy impossible. Instead, they would be forced to resort to the “stepping” strategy commencing only with the ensuing step of the non-amputated limb. On the other hand, when UTA subjects face an outward-directed perturbation while in-stance on the non-amputated side it was our further hypothesis that their dynamic balancing responses would resemble those of the control population.

## Methods

### Participants

Fourteen high-functioning UTA subjects (11 females, 3 males; age: 49.9 ± 12.4 years; body mass: 81.9 ± 15.2 kg; height: 173.7 ± 9.2 cm; left amputations: 9, right amputations: 5; traumatic amputation: 9 subjects, congenital limb loss: 2 subjects) and nine control subjects (2 females, 7 males; age: 46.7 ± 11.9 years, body mass: 79 ± 15 kg, height: 177.3 ± 7.4 cm) without known neurological and muscular-skeletal problems participated in this study. The inclusion criteria for UTA subjects were: unilateral transtibial amputation, at least 10 years of experience using a passive transtibial prosthesis, have had the current prosthesis for at least one year, the same medical doctor and prosthetist checked for socket fitness and alignment, have no problems with the prosthesis, k-levels K3 and K4 [9], have no other muscular-skeletal problems, are able to walk independently without walking aids, and are able to follow instructions. The study was approved by the local ethics committee (number 45/2018) and all participants provided written informed consent.

### Experimental conditions

**Fig. 1:**
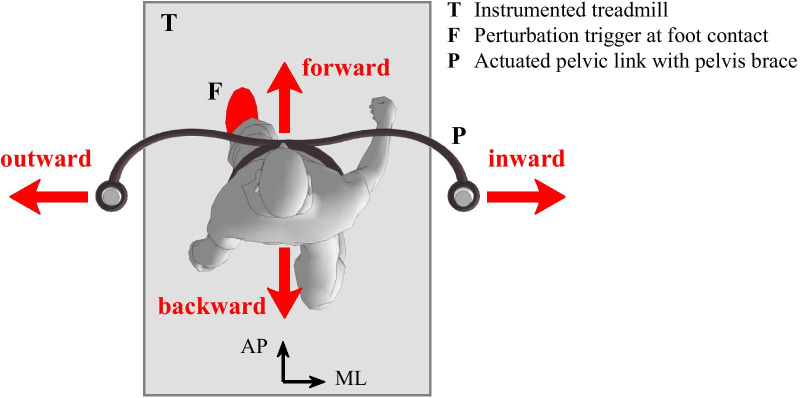
Experimental setup for assessing balance responses after perturbations applied to pelvis. Perturbations were applied in forward, backward, inward, outward directions and were triggered at either left or right foot strike

In this study the Balance Assessment Robot for Treadmill walking (BART), consisting of a wide instrumented treadmill and an actuated pelvic link with pelvis brace [25, 31], was used to deliver perturbing force impulses at the level of the pelvis during walking on the treadmill. Haptic interaction between the actuated pelvic link and the participant’s pelvis, that is in BART implemented via an admittance controller, was in this study set so that the interaction forces between perturbations were as low as possible to allow free pelvis movement. During perturbation period however the admittance controller delivered a force impulse to the pelvis either in forward, backward, inward or outward direction (Fig. 1) at either the left or the right foot contact. The amplitude of perturbation was normalized to 10% of each participant’s body weight and its duration was set to 150 ms as in our previous studies [25, 26]. Pelvis position in the BART was measured with the pelvic link and was used to estimate the movement of center of mass (COM) in a similar way as reported in previous studies [25, 26]. GRF signals were obtained by four precise force transducers (K3D120, ME Systeme GmbH) underneath the treadmill. They were used to determine COP which was passed to the custom developed algorithm on control computer that identified in real time left and right foot strikes. Finally, the treadmill speed was set to 0.5 m/s and was not changed throughout the session.

### Reactive balance assessment protocol

Subjects started with a five-minute introductory session in order to familiarize themselves with the experimental conditions identical to those of the subsequent experiment, i.e. unperturbed and perturbed walking. They were secured with the pelvic brace, which in case of complete loss of balance would hold the subject erect and immediately stop the treadmill. They were instructed to walk within the central area of the treadmill—current pelvic position and the central treadmill area were visualized on a screen in front of the subject for orientation. At the beginning of the experiment each participant walked for approximately three minutes with no perturbations being delivered to assess their unperturbed gait. This initial period was followed by approximately seven minutes of perturbed walking when all test subjects were subjected to seven repetitions for each perturbation direction on both body sides. The time between two consecutive perturbations was randomly chosen within the interval between 6 and 9 s. Altogether each subject received 56 perturbations that were block-randomized. With respect to our hypotheses only dynamic balancing responses following outward-directed perturbed walking were considered in further analysis.

### Measurements and data analysis

The COM, COP and GRF signals were first segmented into gait cycles. Gait cycle was defined as the period between two consecutive foot strikes of the same leg. Gait cycles immediately after the onset of outward-directed perturbation and gait cycles where no perturbation occurred were considered in the analysis. The data of the selected gait cycles were segmented into the “in-stance” periods (from right foot strike to the next left foot strike or from left foot strike to the next right foot strike—from 0% to approx. 60% of gait cycle) and into the “stepping” periods (from left foot strike to the next right foot strike or from right foot strike to the next left foot strike—from approx. 60 to 100% of gait cycle) [44]. In both periods and separately for unperturbed and outward-directed perturbed walking they were normalized to the duration of each period to allow visual comparison between different sub-phases of the in-stance and stepping periods of the gait cycles.

Data were organized into three groups: (i) control group (responses to perturbations delivered at left foot strike in the group of control subjects; as in our previous study [27] we found responses to outward-directed perturbations for left and right side to be comparable), (ii) non-amputated group (responses to perturbations delivered at the foot strike of the non-amputated leg in the group of UTA subjects) and (iii) amputated group (responses to perturbations delivered at the foot strike of the amputated leg in the group of UTA subjects). For each subject COM, COP, GRF and durations of in-stance and stepping periods (T_in-stance_ and T_stepping_ respectively) were averaged across seven repetitions for unperturbed and outward-directed perturbed walking. If any of the seven repetitions markedly differed it was excluded from further analysis. At least five repetitions were averaged for each experimental condition. Peak excursions were obtained from averaged COM and COP for each subject and for outward-directed perturbed walking as well as for unperturbed walking. ΔCOM was calculated as the difference between COM peak excursion of outward-directed perturbed walking and COM peak excursion of unperturbed walking (sagittal plane—ΔCOM_AP_; frontal plane—ΔCOM_ML_). Likewise, ΔCOP was calculated as the difference between COP peak excursion of outward-directed perturbed walking and COP peak excursion of unperturbed walking (frontal plane—ΔCOP_ML_). ΔCOM was determined once in the gait cycle while ΔCOP was determined separately for the in-stance and stepping period of gait cycles. Similarly, for outward-directed perturbed walking we calculated the deviation of durations of in-stance and stepping periods (ΔT_in-stance_ and ΔT_stepping_ respectively) from unperturbed walking.

We have further calculated the time integrals of outward-directed perturbed and unperturbed GRF for the in-stance and stepping periods. Finally, these integrals were normalized to the body mass of each subject and subtracted to yield force impulses for both planes (frontal plane—ΔGRF_ML_) which acted against perturbation in both periods of the gait cycle. ΔCOM can be viewed as a controlled variable while ΔGRF and to some extent also ΔCOP (when the human body can be considered as an inverted pendulum) can be viewed as control variables. Thus, separately determining ΔGRF and ΔCOP for the in-stance and stepping periods provides information on the relative share of dynamic balancing responses coming from the in-stance strategy and stepping strategy.

### Statistical analysis

The normal distribution of data was confirmed using a Kolmogorov–Smirnov test. One-way analysis of variance (ANOVA) was conducted to compare the effect of group factor on T_in-stance_ and T_stepping_ of unperturbed walking in control, non-amputated and amputated groups. Likewise, for outward-directed perturbation one-way ANOVA was conducted to compare the effect of group factor on ΔT_in-stance_ and ΔT_stepping_ in control, non-amputated and amputated groups. Finally, for each combination of outward-directed perturbation and in-stance or stepping periods one-way ANOVA was conducted to compare the effect of group factor (control, non-amputated and amputated) on ΔCOM, ΔCOP and ΔGRF. The Bonferroni method was used in post-hoc comparisons. The level of statistical significance was set to 5%. Data processing and data analysis were performed in MATLAB R2018b (The MathWorks, Inc.).

## Results

### Kinematics and kinetics of perturbed walking

**Fig. 2:**
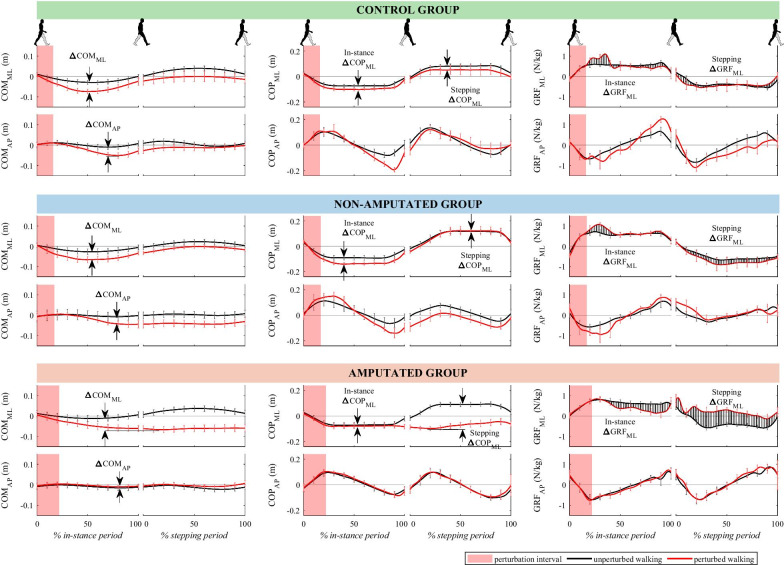
Kinematics and kinetics following outward-directed perturbation. Mean values and standard deviations are shown for the group representatives

Figure 2 shows kinematic and kinetic responses for a representative subject in each of the three groups during unperturbed and perturbed walking. The responses to outward-directed perturbations for a subject from the non-amputated group were similar to those for a subject from the control group. The response was characterized with COP_AP_ displacement under the stance leg toward the toes and with an increase in posterior GRF_AP_ that temporarily decelerated COM_AP_ (braking strategy). COP_ML_ was displaced toward the outer edge of the foot (medio-lateral ankle strategy) whereas an impulse-like increase in lateral GRF_ML_ acted to decelerate COP_ML_ movement (inertial strategy). Perturbation was contained completely by the described in-stance strategy.

On the other hand, in the responses of a subject from the amputated group we noticed a complete lack of in-stance response after outward-directed perturbation. Here the main strategy was first to move the non-amputated leg substantially more laterally to produce a “cross-step”. Once the non-amputated leg entered the stance phase of a gait cycle, which was substantially more laterally compared to unperturbed walking, the established lateral position of COP_ML_ in relation to COM_ML_ modified lateral GRF_ML_ which acted to decelerate COM_ML_ movement. This stepping response resulted in a substantially higher maximal excursion of COM_ML_ in the frontal plane.

### Temporal parameters of unperturbed walking

**Fig. 3:**
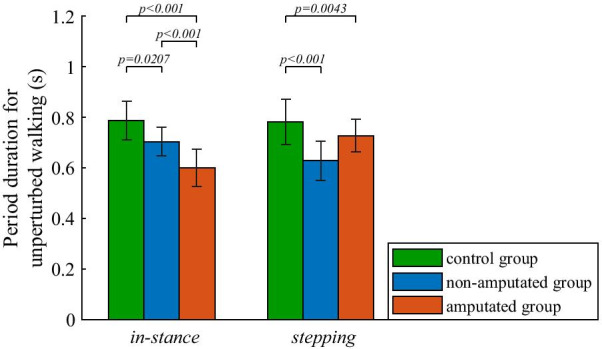
Group mean values and standard deviations for the duration of in-stance and stepping periods of unperturbed walking for all three groups. P-values indicate statistically significant effect of group factor and statistically significant differences between groups in Bonferroni post-hoc paired comparisons were found

Figure 3 shows durations of in-stance (T_in-stance_) and stepping (T_stepping_) periods for all three groups for unperturbed walking. Results show that the control group shared the duration of gait cycles approximately equally between in-stance and stepping periods. On the other hand, UTA subjects shortened the duration of gait cycle (combined T_in-stance_ and T_stepping_) with respect to control subjects and preferred to extend the duration of in-stance period T_in-stance_ and shorten the duration of stepping period T_stepping_ on the non-amputated side. Further statistical analysis showed a statistically significant effect of group factor on T_in-stance_ (F(2,34) = 21.27; p < 0.001) for the three groups and post-hoc analysis showed statistically significant differences in all pair-wise comparisons between groups. The group factor had statistically significant effect also on T_stepping_ (F(2,34) = 12.4; p < 0.001), however only T_stepping_ of the control group with respect to non-amputated and amputated groups was statistically different in the post-hoc comparison.

### Temporal parameters of perturbed walking

**Fig. 4:**
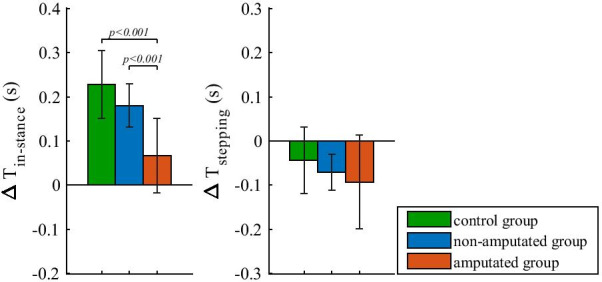
Temporal parameters of unperturbed and outward-directed perturbed walking. Group mean values and standard deviations are shown for all three groups. P-values indicate statistically significant effect of group factor and statistically significant differences between groups in Bonferroni post-hoc paired comparisons were found

Figure 4 shows changes in temporal parameters for the three selected groups after outward-directed perturbation. All groups have shown increased duration of in-stance period with respect to unperturbed walking. There was a statistically significant effect of group factor on ΔT_in-stance_ (F(2,34) = 16.4577; p < 0.001) and post-hoc comparisons showed that ΔT_in-stance_ for the amputated group was significantly smaller than ΔT_in-stance_ for the control and non-amputated groups. On the other hand, after perturbation all groups shortened the duration of stepping period T_stepping_ with respect to unperturbed walking. Group factor did not have significant effect on ΔT_stepping_ (F(2,34) = 1.0512; p = 0.1143).

### ΔCOM of perturbed walking

**Fig. 5:**
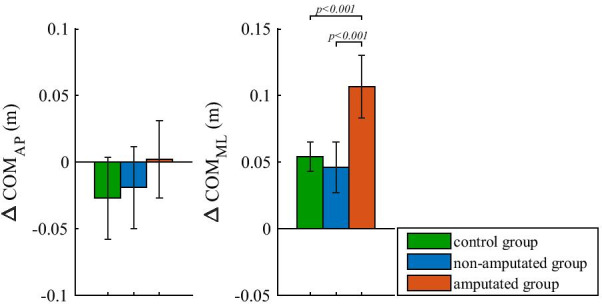
ΔCOM of outward-directed perturbed walking. Group mean values and standard deviations are shown for all three groups. P-values indicate statistically significant effect of group factor and statistically significant differences between groups in Bonferroni post-hoc paired comparisons were found

Figure 5 shows ΔCOM_AP_ and ΔCOM_ML_ for the three selected groups. ΔCOM_AP_ shows a substantial posterior shift of pelvis after outward-directed perturbation for control and non-amputated groups that was more pronounced in the control group. One-way ANOVA showed a significant effect of group factor on ΔCOM_AP_ (F(2,34) = 3.0192; p < 0.001) but no significant differences were found in post-hoc analysis.

In the mediolateral direction ΔCOM_ML_ indicates considerable and almost equal shift of pelvis in the direction of the outward-directed perturbation for the control and non-amputated groups. Even larger—almost double—pelvis shift in the direction of perturbation occurred in the amputated group. Statistical analysis showed that group factor had significant effect on ΔCOM_ML_ (F(2,34) = 38.5987; p < 0.001). Subsequent post-hoc comparison showed that ΔCOM_ML_ for the amputated group was significantly higher than ΔCOM_ML_ for the control group as well as for the non-amputated group.

### ΔCOP and ΔGRF of perturbed walking

**Fig. 6:**
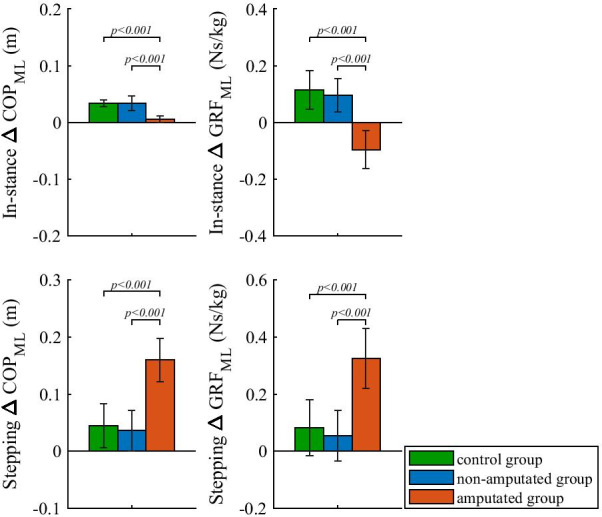
ΔCOP and ΔGRF of outward-directed perturbed walking. Group mean values and standard deviations are shown for all three groups. P-values indicate statistically significant effect of group factor and statistically significant differences between groups in Bonferroni post-hoc paired comparisons were found. Note that positive values of ΔCOP_ML_ indicate change in the direction of perturbation direction. Also note that positive values of ΔGRF_ML_ indicate change in the direction opposite to perturbation direction

Figure 6 shows ΔCOP and ΔGRF for the three selected groups. In the in-stance period ΔCOP_ML_ shows a moderate shift of COP in the outward direction for control and non-amputated groups when compared to unperturbed walking whereas for the amputated group the shift was marginal. Statistical analysis showed a significant effect of group factor on ΔCOP_ML_ in the in-stance period (F(2,34) = 40.1967; p < 0.001) and post-hoc comparison showed that in the in-stance period ΔCOP_ML_ for the amputated group was significantly smaller than ΔCOP_ML_ for control and non-amputated groups.

In addition, ΔGRF_ML_ shows that GRF_ML_ in the in-stance period for the control and non-amputated group increased on average after perturbation when compared to unperturbed walking and decreased for the amputated group. Statistical analysis showed a significant effect of the group factor on ΔGRF_ML_ in the in-stance period (F(2,34) = 42.2117; p < 0.001) and further post-hoc analysis showed that in the in-stance period ΔGRF_ML_ for the amputated group was significantly different than ΔGRF_ML_ for the control and non-amputated group.

In the stepping period ΔCOP_ML_ again shows a moderate shift of COP in the direction of perturbation for control and non-amputated groups when compared to unperturbed walking whereas the shift is substantial for the amputated group. Statistical analysis showed a statistically significant effect of group factor on ΔCOP_ML_ in the stepping period (F(2,34) = 45.3744; p < 0.001) and further post-hoc comparison again showed that in the stepping period ΔCOP_ML_ for the amputated group was significantly larger than ΔCOP_ML_ for control and non-amputated groups.

Finally, ΔGRF_ML_ also shows that GRF_ML_ in the stepping period for the control and non-amputated group slightly increased on average after perturbation when compared to unperturbed walking and increased substantially for the amputated group. Statistical analysis showed that the effect of group factor on ΔGRF_ML_ in the stepping period was statistically significant (F(2,34) = 31.1269; p < 0.001). Post-hoc analysis again showed that in the stepping period ΔGRF_ML_ for the amputated group was statistically larger than ΔGRF_ML_ for control and non-amputated groups.

## Discussion

### Dynamic balancing responses

In this study we investigated dynamic balancing responses following perturbations applied to the pelvis in the outward direction during slow treadmill walking in a group of control subjects and a group of high-functioning UTA subjects. Results show that the control group apply the in-stance balancing strategy, which is consistent with the findings of our previous study [28]. Similarly, when UTA subjects were subjected to outward-directed perturbation upon entering stance phase with their non-amputated side they were also able to modulate COP and GRF in a similar way which indicates they used the in-stance strategy. However, when perturbations occurred when entering the stance phase on the amputated side UTA subjects did not show such COP and GRF modulation under their prosthetic leg which indicates a lack of in-stance balancing strategy. Instead, the balancing response commenced only after the non-amputated leg entered the ensuing stance when it was placed to make a cross-step. This indicates that their response mainly consisted of the stepping strategy. Delaying the corrective action resulted in higher COM displacement, which is in agreement with findings from other perturbation studies conducted in subjects without amputation and post-stroke subjects [12, 26, 44]. These findings support our hypotheses.

However the findings of the present study seems to only partially conform to the results of other studies that have investigated dynamic balancing responses following unexpected perturbing pushes in the frontal plane applied to the pelvis of UTA subjects [23, 24]. Namely UTA subjects in those studies developed stepping responses regardless whether the non-amputated or amputated leg was in stance phase at the time of perturbation [23, 24] whereas in the present study UTA subjects applied in-stance and not stepping strategy when they counteracted outward-directed perturbation on the non-amputated side. Discrepancy between studies is to be attributed to different walking speeds which was in those studies 0.8 m/s whereas in in the present study it was set to 0.5 m/s to match the average walking speed of UTA subjects after discharge [1]. Namely a recent study showed that balancing responses, when evoked by force impulses to the pelvis of subjects without amputation at walking speeds of 0.8 m/s and higher, predominantly consist of stepping responses while the contribution from in-stance responses is small [27]. However, when the speed of walking is low (0.4 to 0.6 m/s) in-stance strategies are the primary mechanisms of dynamic responses in persons without amputation [27].

The results of this study imply that the lack of braking strategy in the sagittal plane due to missing ankle efferent input in UTA subjects may interfere also with the development of balancing strategies in frontal plane. Namely, our previous study [28] shows that when subjects without amputation were subjected to outward-directed perturbation while walking very slowly inertial strategy (modulation of lateral component of GRF) and braking strategy (modulation of posterior component of GRF) are closely coupled and that this coupling is necessary to efficiently control angular momenta in the frontal and sagittal planes [28]. Such connections was found also in UTA subjects where reduced braking on the amputated side was associated with a significantly larger range of angular momentum in the sagittal plane in the first half of the gait cycle and that the absence of braking was a compensatory mechanism that helped to control angular momentum in the sagittal plane [40].

Our study also shows that even though the treadmill speed was set to be equal for all subjects, gait cycle time during unperturbed walking was significantly shorter for UTA subjects than in control subjects. This implies that cadence was higher and steps were for UTA subjects shorter. They also spent considerably more time in stance on the non-amputated side than on the amputated side. This is in agreement with findings of other studies that also reported of such proactive behaviour [2, 13, 23, 24, 34, 37] and may be related to the potential need of being capable to react faster to prospective perturbation with ensuing step. Since the perturbations in our study occurred at the beginning of the stance phase, adopting shorter and faster steps enabled the group of UTA subjects to react faster with the ensuing step of the non-amputated leg.

### Clinical relevance

The perturbing paradigm used in this study bears close similarity with other perturbing modalities that one may come across during the activities of daily living. Perturbing pushes to the pelvis of a walking subject in the outward direction mimic situations where persons collide with other persons in a crowd. Moreover, experiments where platform movement was used to mimic slips have shown that perturbations in the form of a slip show certain degree of similarity to perturbations in the form of waist pushes [15]. In this context results of this study suggest that the lack of dynamic response in counteracting a perturbing push when applied at the beginning of stance phase on the amputated side may also be an indication of potentially unreliable responses to collisions and slips in everyday walking that may often result in falls. Namely, about 50% of persons with amputation fall at least once a year, almost 10% of them fall due to COM disruption, 18% fall due to slip and 16% fall due to trip [21]. This should be further investigated in the future. This also shows the importance of proper control of COM position with respect to the base of support as significant displacement of COM from the base of support, resulting from sensory fusion errors in the internal balance model, without in-stance mechanisms to mitigate the effect of such gait instability inherently increases the risk of falling. To improve the dynamic balance in such situations rehabilitation after lower limb amputation should also incorporate task-oriented perturbation training as part of the rehabilitation program. The benefits of perturbation-based training in a virtual environment [38] and on a moving platform [19] have already been shown: it improves mediolateral stability, decreases step width [38] and reduces the incidence of falls [19]. Therefore it is reasonable to expect that perturbation training at slow walking speed would also improve reactive responses as well as overall gait characteristics and make UTA subjects better prosthesis users.

### Methodological considerations and limitations of the study

In this study a relatively low walking speed of 0.5 m/s was selected for assessment of dynamic balancing responses to match average walking speed of UTA subjects after discharge [1]. This created conditions in which subjects without amputation typically apply in-stance balance strategies when subjected to outward-directed perturbations. A recent study with subjects without amputation shows that when walking on a treadmill with walking speeds ranging from 0.4 to 0.6 m/s, in-stance balancing strategies play the dominant role when counteracting perturbations. Exceeding this range of walking speeds first displays mixing of in-stance and stepping strategies whereas at 0.8 m/s and above only the stepping strategy is used [27].

We also focused on balancing responses to only one perturbation amplitude, i.e., 10% of body weight. Previous studies [16, 27] have shown that such perturbation amplitude is strong enough to elicit substantial imbalance during walking without exciting undesirable leg pivoting or arm and trunk movement. Similarly, setting the onset of perturbation to the beginning of stance phase was motivated by the observation that it elicits the use of the in-stance strategy to the largest extent [27]. It is our opinion that the selected experimental parameters (speed of walking, perturbation amplitude and onset of perturbation) determined experimental conditions in a way that challenged control and UTA subjects to the extent that allowed us to adequately test the posed hypotheses.

The BART was controlled such that the interaction forces between the walking subject and the pelvis link were as low as possible. We have assessed the effect of interaction forces in a previous study and found that the influence of these forces on COP and GRF in the sagittal and frontal planes as well as on the EMGs of major lower limb muscles during unperturbed walking with walking speeds ranging from 0.4 to 0.8 m/s was negligible [32]. In another study we have demonstrated that interaction between the balance assessment robot and the pelvis of a walking subject is purely passive, meaning that there is no exchange of energy between the walking subject and the BART except for the period when a perturbing push is delivered [25]. Thus, we may consider that using the BART to deliver perturbations to the pelvis of walking subjects had negligible effects on the presented results.

Only high-functioning UTA subjects that were experienced walkers have been included in this study. Although the results of this study cannot be directly applied to less experienced UTA subjects it is reasonable to assume that experimental condition as described in this study would demand greater effort that would compel less experienced UTA subjects to rely on the non-amputated side even more. This needs to be further investigated in the future.

In this study the number of female UTA subjects exceed the number of male UTA subjects and the number of male control subjects exceeds the number of female control subjects. Qualitative comparison showed that male and female subjects responded to outward perturbations in similar manner. The potential statistical effect of unequal gender representation was not analysed as it was not the scope of this study nor is it typically investigated in related studies.

## Conclusion

In this study we explored the selection of balancing strategy in UTA subjects after they were subjected to outward-directed perturbations at slow walking. Results show that UTA subjects used similar in-stance balancing strategies as persons without amputation only when perturbation occurred at the heel strike of the non-amputated side. However, as in-stance strategies rely heavily on the ankle motor function, which a resource missing in UTA subjects in-stance strategies did not constitute their dynamic balancing response after the perturbation occurred at the heel strike of the amputated side. Instead they implemented alternative strategy where the response began with a substantial delay and concentrated on stepping strategy. The alternative stepping response is much less efficient or not sufficiently developed to fully substitute the missing in-stance strategies which may be one of the potential causes of frequent falls among the UTA population.

## Data Availability

The data used in this study may be available by the corresponding author upon a reasonable request to any qualified researcher.

## Abbreviations

UTA: Unilateral transtibial amputation
COP: Center of pressure
COM: Center of mass
GRF: Ground reaction force
DoF: Degrees of freedom
ΔCOM: Difference in peak values of COM between perturbed and unperturbed walking
ΔCOM_AP_: Difference in peak values of anteroposterior component of COM direction between perturbed and unperturbed walking
ΔCOM_ML_: Difference in peak values of mediolateral component of COM direction between perturbed and unperturbed walking
ΔCOP: Difference in peak values of COP between perturbed and unperturbed walking
ΔCOP_AP_: Difference in peak values of anteroposterior component of COP between perturbed and unperturbed walking
ΔCOP_ML_: Difference in peak values of mediolateral component of COP between perturbed and unperturbed walking
T_in-stance_: Duration of in-stance period
T_stepping_: Duration of stepping period
ΔT_in__-stance_: Difference in duration of in-stance period between perturbed and unperturbed walking
ΔT_stepping_: Difference in duration of stepping period between perturbed and unperturbed walking
ΔGRF: Difference in GRF integrals between perturbed and unperturbed walking
ΔGRF_AP_: Difference in anteroposterior component of GRF integrals between perturbed and unperturbed walking
ΔGRF_ML_: Difference in mediolateral component of GRF integrals between perturbed and unperturbed walking
